# Gold Nanoparticles as a Direct and Rapid Sensor for Sensitive Analytical Detection of Biogenic Amines

**DOI:** 10.1186/s11671-017-2014-z

**Published:** 2017-03-29

**Authors:** K. M. A. El-Nour, E. T. A. Salam, H. M. Soliman, A. S. Orabi

**Affiliations:** 1 Present Address: Department of Chemistry, College of Liberal Arts and Science, University of Florida, Gainesville, FL 32611-7200 USA; 2 Department of Chemistry, Faculty of Science, Suez Canal University, Ismailia, 41522 Egypt

**Keywords:** Histamine, Biogenic amines, Gold nanoparticles, Spoilage marker, Colorimetric sensor

## Abstract

A new optical sensor was developed for rapid screening with high sensitivity for the existence of biogenic amines (BAs) in poultry meat samples. Gold nanoparticles (GNPs) with particle size 11–19 nm function as a fast and sensitive biosensor for detection of histamine resulting from bacterial decarboxylation of histidine as a spoilage marker for stored poultry meat. Upon reaction with histamine, the red color of the GNPs converted into deep blue. The appearance of blue color favorably coincides with the concentration of BAs that can induce symptoms of poisoning. This biosensor enables a semi-quantitative detection of analyte in real samples by eye-vision. Quality evaluation is carried out by measuring histamine and histidine using different analytical techniques such as UV–vis, FTIR, and fluorescence spectroscopy as well as TEM. A rapid quantitative readout of samples by UV–vis and fluorescence methods with standard instrumentation were proposed in a short time unlike chromatographic and electrophoretic methods. Sensitivity and limit of detection (LOD) of 6.59 × 10^−4^ and 0.6 μM, respectively, are determined for histamine as a spoilage marker with a correlation coefficient (*R* ^2^) of 0.993.

**Electronic supplementary material:**

The online version of this article (doi:10.1186/s11671-017-2014-z) contains supplementary material, which is available to authorized users.

## Background

Poor storage of meat and poultry meat as well as their products caused by upkeep at inappropriate temperatures results in meat decomposition by pathogenic microorganisms. Acting these pathogenic microorganisms produces harmful amines which cause food poisoning [1]. These biogenic amines (BAs), result from bacterial decarboxylation of amino acids, could not be outstanding by smelling the odor because they are created even in meat preserved at 5 °C for more than 10 days. BAs can be used as a spoilage marker of meat, poultry meat, and their products [2, 3]. High levels of biogenic amines such as histamine, tyramine, phenylethylamine, and cadaverine can be used as a signal of hygienic food quality as they possibly can cause food poisoning [4, 5]. Histamine forms in poultry meat by decarboxylation of the amino acid histidine catalyzed by l-histidine decarboxylase in the presence of decarboxylase-positive microorganisms [6]. In the human body, the biogenic amines affect many systems such as the respiratory system, digestion system, and heart [7]. Additionally, biogenic amines with secondary amine groups can produce nitrosamines when reacting with nitrites and hence have cancer-producing ability. Previously, several studies have determined biogenic amines in different types of food [8–12]. In another study, histamine was follow to detect the freshness of fish which was rich in histidine [13].

Most biogenic amines concentrations increase as the storage time increases, so it could be used as a good marker for the life time and freshness of food. Many different methods involving histamine determination have already been explained [14]. In particular, a spectrofluorimetric method was used for histamine level determination in canned fish [15]. Also, high-performance liquid chromatography (HPLC) [16], capillary electrophoresis (CE) [17], gas chromatography along with mass spectrometry (GC-MS) [18], and thin-layer chromatography (TLC) were described for BA determinations [19]. Time needed for analysis of biogenic amines using those techniques ranges from 45 to 150 min per sample. Many disadvantages of using the HPLC, CE, GC-MS, and TLC analyses in general are (i) the long time needed for sample pretreatment and analysis, (ii) the requirement of using organic solvents of high quality, which is quite expensive, and (iii) the disposal of the used organic solvents has to be taken into consideration. On the other hand, other methods were used for BAs detection such as disposable screen-printed electrode biosensors with enzymes [20]. These biosensors have an advantage of reducing sample pretreatment. Another approach uses a home-built reflectometric sensing system to monitor the total volatile amines [21, 22], but the calibration device for these sensors is not easily available in labs. Hence, fast and sensitive detection of BAs as food spoilage marker is needed.

Recently, applying nanosensors in a variety of fields such as medical for cancer diagnosis and treatment, biological, chemical, and food industry have elevated [23–26]. Within the food industry where measuring the food quality is expounded straight to the general public health, development of nanosensors, for food safety examination, becomes needful [27–31]. Nanosensors have the advantage of efficient detection for pathogen rapidly with high sensitivity [32–34]. Also, they function as “electronic noses” by detecting chemicals released during food spoilage [35–38].

Gold nanoparticles (GNPs) have acquired much attention as a biosensor simply because they have a lot of intriguing qualities [37, 38], which enable them to be used as signal amplification tags in diverse biosensors [39–41].

In this work, we developed a sensing tool for the quantitation of biogenic amines in real samples (e.g., poultry meat) as a rapid screening tool compared to HPLC or other more time-consuming methods. The new sensing method using gold nanoparticles enables rapid detection of BAs (even by visible readout) with standard fluoremetric and spectrophotometric means for direct determination of histidine and histamine as biomarkers for freshness and spoilage of poultry meat with high sensitivity.

## Methods

### Materials

Histamine dihydrochloride (C_5_H_9_N_3_·2HCl), histidine (C_6_H_9_N_3_O_2_), tetrachloroauric acid (HAuCl_4_), trisodium citrate (Na_3_C_6_H_5_O_7_), and NaCl were purchased from Sigma (St. Louis, MO, USA) (http://www.sigmaaldrich.com). Chicken breast samples were purchased from a local retail store.

#### Samples Preparation

Two samples from chicken white meat were taken, one is considered as a fresh sample (FS) and yet another one remained at 4 °C for 15 days and is considered as a spoiled sample (SS). Each sample is fragmented into three equal parts of weight (5 g). Each sample was mixed with 50 mL of 0.9% saline (NaCl) solution and were subjected to homogenization for 1 h, then filtered off. A centrifugation from the homogenized solution was done to each sample at 3500 rpm for half an hour. Each sample was then collected in 50 mL bottle and kept at −18 °C.

#### Preparation of Amine Working Solutions

Amines standard solutions were made by dissolving a quantity of every amine (histamine and histidine) in 50 mL using 0.9% saline solution (NaCl) to acquire solutions with concentrations of 0.6, 2, 6, 10, 14, and 18 μM, correspondingly. Solutions were freshly daily prepared, and experiments were performed at room temperature.

#### Preparation of Gold Nanoparticles (GNPs)

All the glass wares were cleaned with nitric acid and washed with double distilled water before use. GNPs were prepared as discussed previously [42]. Briefly, 125 mL of deionized water were poured into a 250-mL flask and heated until boiling, then 2 mL of 1% tetrachloroauric acid solution was added and the solution was stirred for 2 min. Ten milliliters of 0.05 M sodium citrate was gradually added with continuous stirring and heating till the color of the solution changed from faint yellow to deep red at about 10 min indicating the formation of GNPs. The gold nanoparticles were gradually formed as the citrate reduces Au(III) to Au(0) as indicated by the red color appearance. GNP solution was cooled down at room temperature and stored at 4 °C.

The stability study of the gold nanoparticles over time (from 1 to 10 days) was monitored using absorption spectroscopy at room temperature. The analysis of the characteristic absorption peak *λ*_max_ and Δ*λ* over a 10-day period was checked for the precipitation of GNPs.

#### Preparation of Histidine–GNP and Histamine–GNP Composites

GNPs–histidine and GNPs–histamine solutions were prepared by mixing GNP solution with histamine and histidine standard solutions of concentrations 0.6, 2, 6, 10, 14, and 18 μM, each mixture was stirred for 15 min. The solutions of GNPs with fresh and spoiled chicken samples were prepared by mixing GNPs with (FS) and (SS) in saline. The mixtures were stirred for 15 min.

### Instruments

Morphology of GNPs, GNPs–histamine, and GNPs–histidine were studied by subjecting to high-resolution transmission electron microscope (TEM) using JEOL JEM 2100 (Japan). The interaction of GNPs with histamine and histidine was studied by UV–visible spectra at room temperature with samples in 1 cm quartz cuvette using a SHIMADZU UV 1800 spectrophotometer. Fluorescence spectra were also recorded at room temperature with samples in a quartz cuvette using a Jasco FP 6300 Spectrofluorometer. Fourier transform infrared spectroscopy (FTIR) was recorded at room temperature using a BRUKER TENSOR 27 ratio recording infrared spectrophotometer.

## Results and Discussion

### GNPs Formation and Characterization

The colloidal gold is formed since the citrate ions act as both reducing and capping agents. Because the citrate molecules settle on the particle surface, GNPs are stabilized through electrostatic interaction [43]. Forming gold nanoparticles was preliminarily confirmed by visual observation of color change from pale yellow to deep red color. Recent reports have proven the color is due to the collective oscillation from the electrons within the conduction band, referred to as surface plasmon oscillation. The oscillation frequency is generally within the visible region for gold inducing the strong surface plasmon resonance absorption [44].

**Scheme 1:**
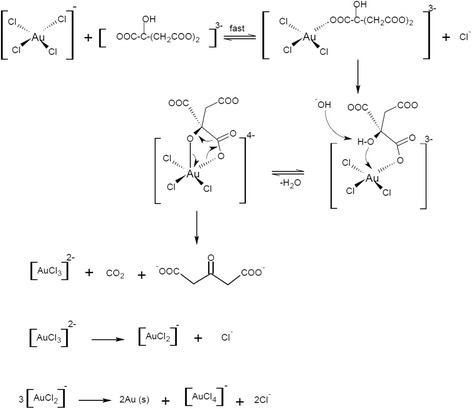
Formation mechanism of GNPs

Reducing tetrachloroauric acid with sodium citrate to form GNPs is illustrated in Scheme 1 [45]:

In Scheme 1, Au(III) is reduced to Au(I) which would involve two steps: (a) a fast ligand exchange with the citrate anion to form an intermediate complex and (b) an equilibrium to give a ring closure followed by a slow and rate determining step involving a concerted decarboxylation and the reduction of Au(III) species. Thus, the citrate anion is anticipated to coordinate equatorially substituting a planar Cl¯ ligand and forming the related complex (Scheme 1). Deprotonation of alcohol group and coordination of the alcohol oxygen axially to Au(III) to provide a pentacoordinated intermediate complex that takes place like a rapid equilibrium adopted by axial complex splintering into products in the rate-limiting step (Scheme 1).

#### Transmission Electron Microscopy (TEM)

**Fig. 1:**
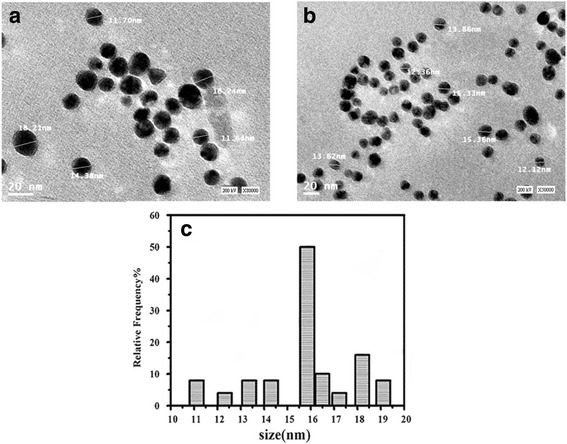
**a**–**c** TEM micrograph of GNPs and the size distribution histogram of GNPs

TEM measurements were carried out to determine the morphology and shape of the formed NPs. Micrograph of GNPs represented in Fig. 1 revealed that they are spherical and well dispersed without agglomeration. Figure 1c shows the representative nanoparticle size histograms of gold nanoparticles. The majority of nanoparticles were between 11 and 19 nm in dimensions and have an average size of 16 nm.

#### UV–vis and Fluorescence Spectroscopy

The produced nanoparticles were subjected to characterization by UV–vis spectroscopy. Sharp peak peak provided by UV–vis spectrum at 520 nm confirms the nanoparticle formation [46]. The particle concentration of the GNPs (15 nM) was determined based on Beer’s law utilizing a molar extinction coefficient of 2.43 × 10^8^ M^−1^ cm^−1^ [39].

The fluorescence of the prepared gold nanoparticles implies that GNPs excited at *λ*_exc_ 540 nm and display emission band at *λ*_emi_ 778 nm.

#### FTIR Spectroscopy

**Scheme 2:**
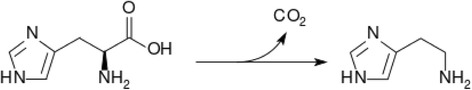
Formation of histamine by histidine decarboxylation

FTIR spectrum of GNPs represent several groups of lines associated with the citrate molecules linked with the surface of gold nanoparticles. A well-developed peak centered at 3715 cm^−1^ might be assigned as *ν*_sym_ and *ν*_asym_ of H_2_O molecule and could be accompanied by O–H stretching of the citrate group. The H_2_O moiety also gave bending vibrational band at 1619 cm^−1^. The bands which appeared at 1385 and 1319 cm^−1^ might be assigned as *ν*_asym_ and *ν*_sym_ of the COO^−^ group. Δ*υ* = |*υ*_asym_ − *υ*_sym_| = 66 cm^−1^ revealed the monodentate interaction of the COO^−^ group [47]. Also, the peak which appeared at 1065 cm^−1^ might be raised from the Au-citrate compound [43] (Scheme 2).

### GNPs Sensing Sensitivity of histidine and histamine

The sensing sensitivity of GNPs for measurement of histidine as a natural occurring amine present in chicken meat protein is evaluated. Histamine resulting from bacterial decarboxylation of histidine leading to denaturation of the protein suggesting chicken meat spoilage is also measured. Determination of histidine and histamine is made by addition of GNPs before measuring the real fresh and spoiled chicken samples. The color variations were characterized using vision readout, TEM images, UV–vis, fluorescence, and FTIR spectroscopy.

#### Detection of Histidine–GNPs and Histamine–GNPs

No color change of GNP solution was observed after adding histidine. The color of histamine without GNPs is colorless, but on inclusion of GNPs to various concentrations of histamine, a faint blue color remarked which turns to dark blue with increasing of histamine concentration (0.6–20 μM). The change of color is distinct enough even to enable a semi-quantitative sample readout on potential toxicity (existence of histamine) by eye-vision.

Absorption of light by the surface plasmons of small metallic particles accounts for the colorful appearance of suspensions of those particles. The small size of nanoparticles enables them to bind to target analyte, significantly affecting their optical properties [48], so the color is affected by the size and the extent of aggregation of the particles [49–51]. Attaching histamine on gold nanoparticles is expected to affect surface plasmons which results in changes in the solution color due to particle aggregation.

#### TEM Analysis

**Fig. 2:**
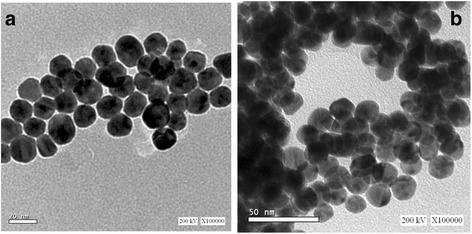
TEM micrograph of **a** histidine–GNPs and **b** histamine–GNPs

TEM micrograph of histidine–GNPs and histamine–GNPs (Fig. 2) show the particle aggregation for histamine–GNPs despite the fact that nanoparticles still spherical. The precise reasons for the aggregation have not been established; however, they likely involve hydrophobic interaction, in the same manner as was observed for elastin bound to gold nanoparticles [52, 53].

#### UV–vis Spectroscopy

UV–visible spectra of GNPs with various concentrations of histidine and histamine show red shifts in absorption peaks of histidine and histamine in contrast to their absorption spectra which was reported before (Additional file 1) [53]. Peaks shift to 216, 215, and 529 nm for histidine, histamine, and GNPs, respectively. These bathochromic shifts in the surface plasmon absorption maxima, which result from changes in the electron density on the surface, may confirm the formation of chemically bonded histidine–GNP and histamine–GNP composites.

#### Fluorescence Spectra

**Fig. 3:**
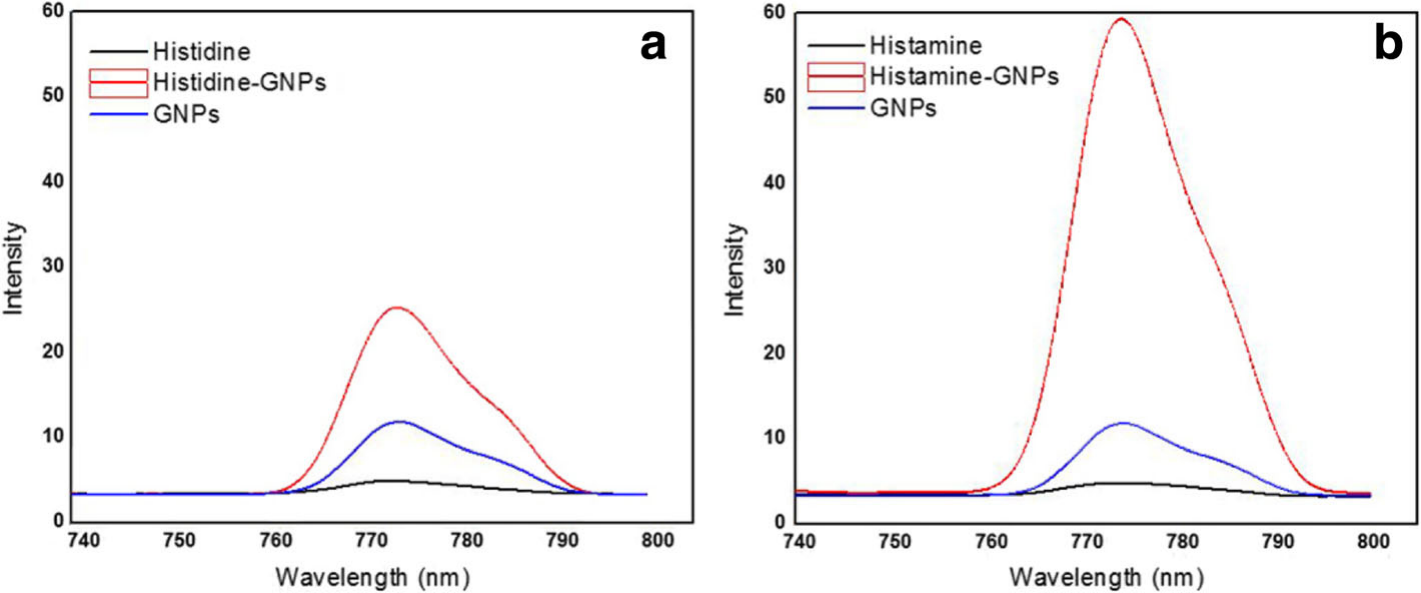
Fluorescence spectra of **a** histidine–GNPs and **b** histamine–GNPs

The histidine and histamine are electronically excited at *λ*_exc_ = 470 nm and gave an emission band centered at *λ*_emi_ = 778 nm (Fig. 3a, b). Forming histidine–GNPs and histamine–GNPs improves the intensity of the emission band and provides a great indication about using GNPs like a biosensor for detection of histamine.

The hyperchromic effect in the emission band of histamine–GNPs are closely related to developing large clusters due to effective aggregations associated with red shift [48].

#### FTIR Spectral Results

The obtained FTIR spectral data are summarized in Table 1. The results revealed the formed composites of histidine–GNPs and histamine–GNPs are significantly different than that of histidine and histamine studied previously [54, 55]. This difference gives strong evidence on forming composites. The strong and broad band which appeared in the 3200–3600 cm^−1^ range (figure is not shown) could be assigned as the stretching vibration of the H_2_O molecules, NH_3_^+^, NH^+^, NH, and COOH moieties. The presence of NH_3_^+^ could be confirmed by the band which centered at 2150 cm^−1^ which assigned as the stretching vibration of the amine hydrochloride. The strong band appeared at 1640 cm^−1^ for histidine molecule could be assigned as the *ν*_as_COO^−^ and *δ*_as_NH_3_^+^; meanwhile, the weak band (shoulder) which appear at 1410 cm^−1^ could be assigned as ν_sym_ COO^−^. Shift in the *ν*_as_ and *ν*_sym_ of the COO^−^ group in case of the histidine–GNP composite point to interaction of the COO^−^ and NH_3_^+^ groups with the GNPs [54, 55].

**Table 1:** FTIR bands of histidine, histidine–GNPs, histamine, and histamine–GNPs

	νH_2_O/νNH/νNH_2_ and νCOOH	νamine-HCl	ν_asy_COO^−^, δ_asy_NH, and δNH_3_^+^	ν_sym_ COO^−^, δ_sym_ NH, and νC=N
Histidine	3300–3600	2150	1640	1410
Histidine–GNPs	3400	2077	1635	-
Histamine	3100	2062	1642	1508
Histamine–GNPs	3300–3500	2142	1545	1436

**Scheme 3:**
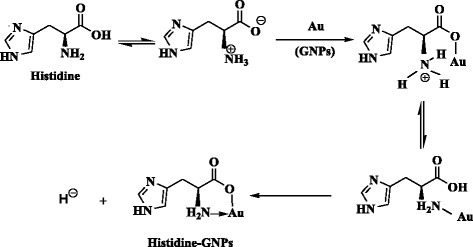
Interaction between GNPs and histidine to form histidine–GNPs

**Scheme 4:**
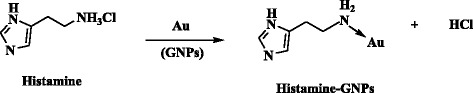
Interaction between GNPs and histamine to form histamine–GNPs

The strong band which centered at 3100 cm^−1^ from histamine could be assigned as *υ*(NH_2_) and *υ*(NH) stretching vibration bands. Also, a broad band which appeared at 3300–3600 cm^−1^ could be assigned as *ν*_sym_H_2_O and *ν*_as_H_2_O. Also, histamine gave bands centered at 2062 cm^−1^ due to the stretching vibration of the amine hydrochloride. The bands centered at 1642 and 1508 cm^−1^ could be assigned as *δ*(NH) and *υ*(C=N) frequencies. The shape and position of the bands of *υ*(NH) gave some change in histamine–GNP composite which revealed forming the histamine–GNP system [54, 56]. So, according to the FTIR spectra, the reaction of histidine and histamine with GNPs could be summarized in Schemes 3 and 4.

### Validation of the Obtained Results

**Fig. 4:**
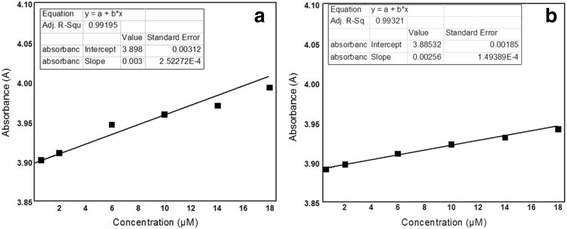
The calibration curve of **a** histidine–GNPs and **b** histamine–GNPs at *λ*_max_ 527 nm

Our results obtained by using GNPs as an optical sensor for detection of histamine as a spoilage marker in rotten chicken meat show good sensitivity and limit of detection (LOD). Absorbance of different concentrations of histidine-GNPs and histamine–GNPs was measured by UV–vis, and the relationship between concentration and absorbance was plotted as shown in Fig. 4. The sensitivities of measurements of histidine–GNPs and histamine–GNPs are found to be 7.70 × 10^−4^ and 6.59 × 10^−4^, respectively. Also, LOD of both histidine and histamine is found to be 0.6 μM with a good correlation coefficient of 0.993. The observed linear dynamic range of histamine is from 0.6 to 12 μM.

### Screening of Histidine and Histamine in Real Chicken Meat Samples

**Fig. 5:**
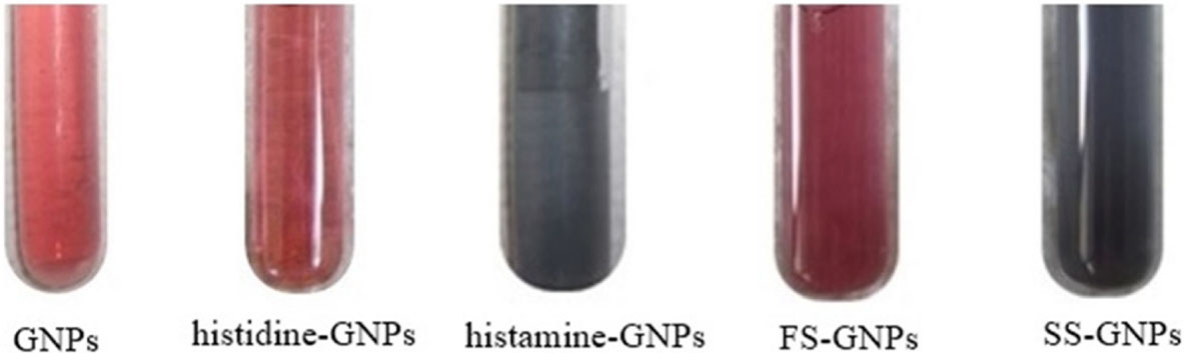
Interaction between GNPs with fresh chicken meat sample (FS) and spoiled sample (SS)

The extracts of the real fresh chicken meat sample (FS) and the spoiled sample (SS) were subjected to interact with GNPs forming FS–GNP and SS–GNP composites (Fig. 5). Color change was observed and investigated using UV–vis and fluorescence as well as FTIR spectra.

On forming FS–GNP composite, a red color was obtained while because of forming SS–GNPs, and an instant deep blue color was obtained which is due to particle aggregation.

**Fig. 6:**
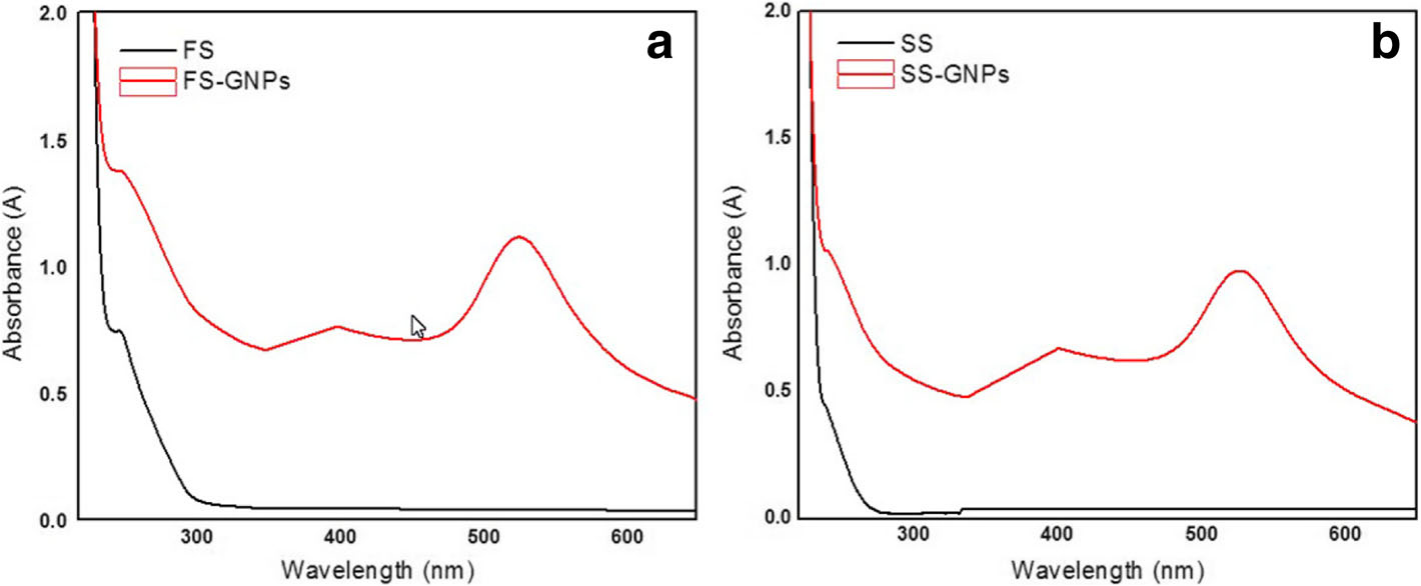
UV–Vis spectra of **a** FS, FS–GNPs and **b** SS, SS–GNPs

UV–vis spectra of FS and FS–GNPs show peaks at 215 and 250 nm corresponding to histidine existing in chicken meat and other species found in the matrix. After addition of GNPs, the spectrum of FS–GNPs show increase in the intensity of peaks at 221 and 527 nm. Also, another broad peak is observed at 400 nm which may be due to matrix in the sample (Fig. 6a).

In SS (Fig. 6b), a peak at 212 nm is observed which may be due to the existence of histamine. On adding GNPs to the SS, a small shift and increase in the intensity of the peak at 217 nm is noticed with the appearance of absorption peak corresponds to GNPs at 527 nm and a peak at 400 nm due to matrix. The remarked shifts in the values of *λ*_max_ are due to chemically bonded molecules, histidine–GNPs, and histamine–GNPs, which induce in the electron density on the surface which results in a shift in the surface plasmon absorption maximum.

**Fig. 7:**
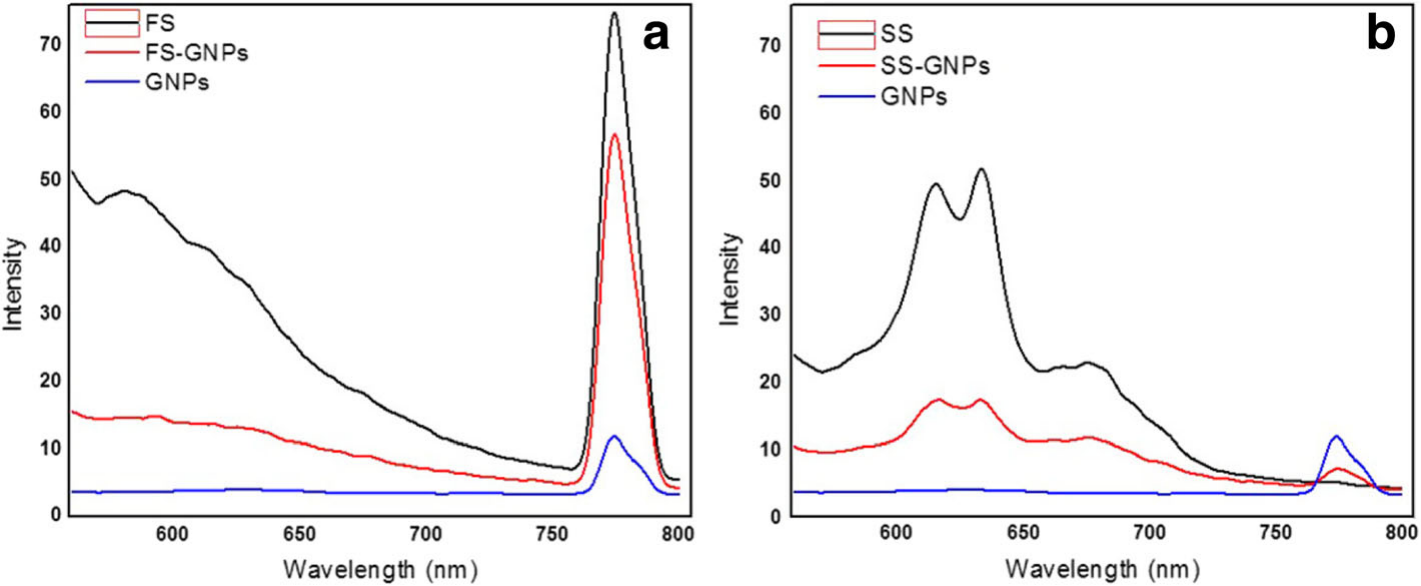
Fluorescence spectra of **a** FS–GNPs and **b** SS–GNPs

The FS moiety was electronically excited at wavelength (*λ*_exc_) = 470 nm and gave emission bands centered at wavelength (*λ*_emi_) = 650 and 778 nm (Fig. 7a). On forming FS–GNPs, a quenching in the intensity of these two bands is noticed. The SS moiety was electronically excited at wavelength (*λ*_exc_) = 470 nm and gave emission bands centered at wavelength (*λ*_emi_) = 620, 640, and 680 nm (Fig. 7b). Upon the formation of SS–GNPs, a quenching in the intensity of these bands is also noticed. Meanwhile, a peak at *λ*_emi_ 778 nm is observed due to GNPs, GNPs-histidine, and GNPs-histamine. The other noted emission bands in the range 620–680 nm may be due to the matrix exist in the real chicken meat samples.

**Fig. 8:**
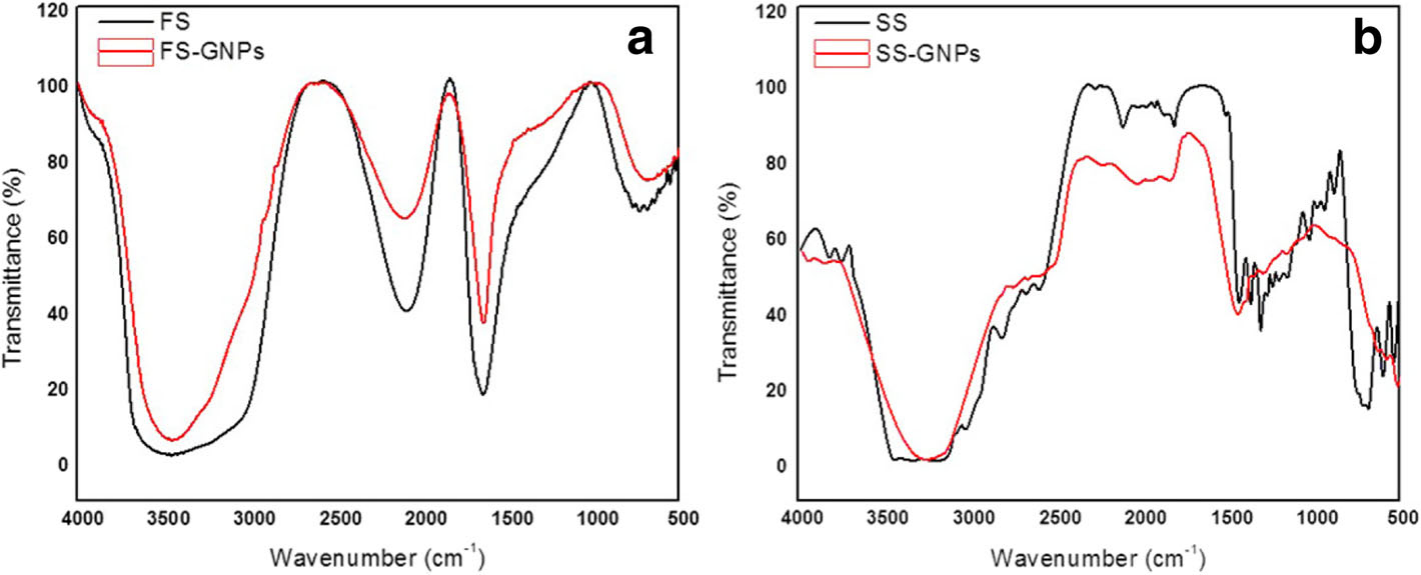
FTIR spectra of **a** FS and FS–GNPs and **b** SS and SS–GNPs

From the FTIR results shown in Fig. 8 and listed in Table 2 of FS, SS, FS–GNPs, and SS–GNPs, the same interpretation is supposed for the existence of histidine as a major component in FS and histamine as a major component in SS. The strong and broad band which appeared at 3000–3600 cm^−1^ could be assigned as *υ*(H_2_O), *υ*(NH), and *υ*(COOH) moieties. The presence of NH_3_^+^ could be confirmed by the band which centered at 2088 cm^−1^ which assigned as the stretching vibration of the amine hydrochloride. The strong band which appeared at 1642 cm^−1^ for FS could be assigned as the *ν*_as_ COO^−^ and δ_sym_ NH_3_^+^; meanwhile, the weak band (shoulder) which shown at 1410 cm^−1^ could be assigned as *ν*_sym_ COO^−^. The *ν*_as_ and *ν*_sym_ bands of the COO^−^ undergo some shift in case of the FS–GNP composite which suggest interacting the COO^−^ and NH_3_^+^ groups with the GNPs [54].

**Table 2:** FTIR bands of FS, FS–GNPs, SS, and SS–GNPs

Vibration type cm^−1^	*ν*(H_2_O), *ν*(NH_3_^+^_,_ NH_,_ NH^+^, NH_2_), and (COOH)	*ν*(amine-HCl)	*ν*_asym_ (COO^−^), *δ*_asym_ (NH_3_^+^) and *δ*_asym_ (NH), *ν*_asym_ (C=N)	*ν*_sym_ (COO^−^), *δ*_sym_ (NH_3_^+^) and *δ*_sym_ (NH), *ν*_sym_ (C=N)
Compounds				
FS	3000–3600	2088	1642	1410
FS–GNPs	3000–3600	2091	1636	-
SS	3000–3600	2160	1434	1367
SS–GNPs	3000–3600	2043	1442	1382

The strong band which centered at 3100 cm^−1^ from SS could be assigned as NH_2_ and NH stretching vibration band. The broad band which appeared in the 3000–3600 cm^−1^ range could be assigned as *ν*_sym_H_2_O and *ν*_as_H_2_O. Also, SS gave a band at 2160 cm^−1^ due to the stretching vibration of the amine hydrochloride. The bands at 1434 cm^−1^ could be assigned as *ν*_sym_(COO^−^). The shape and position of the bands due to NH gave some change in histamine–GNP composite which revealed forming the histamine–GNP system [54].

**Scheme 5:**
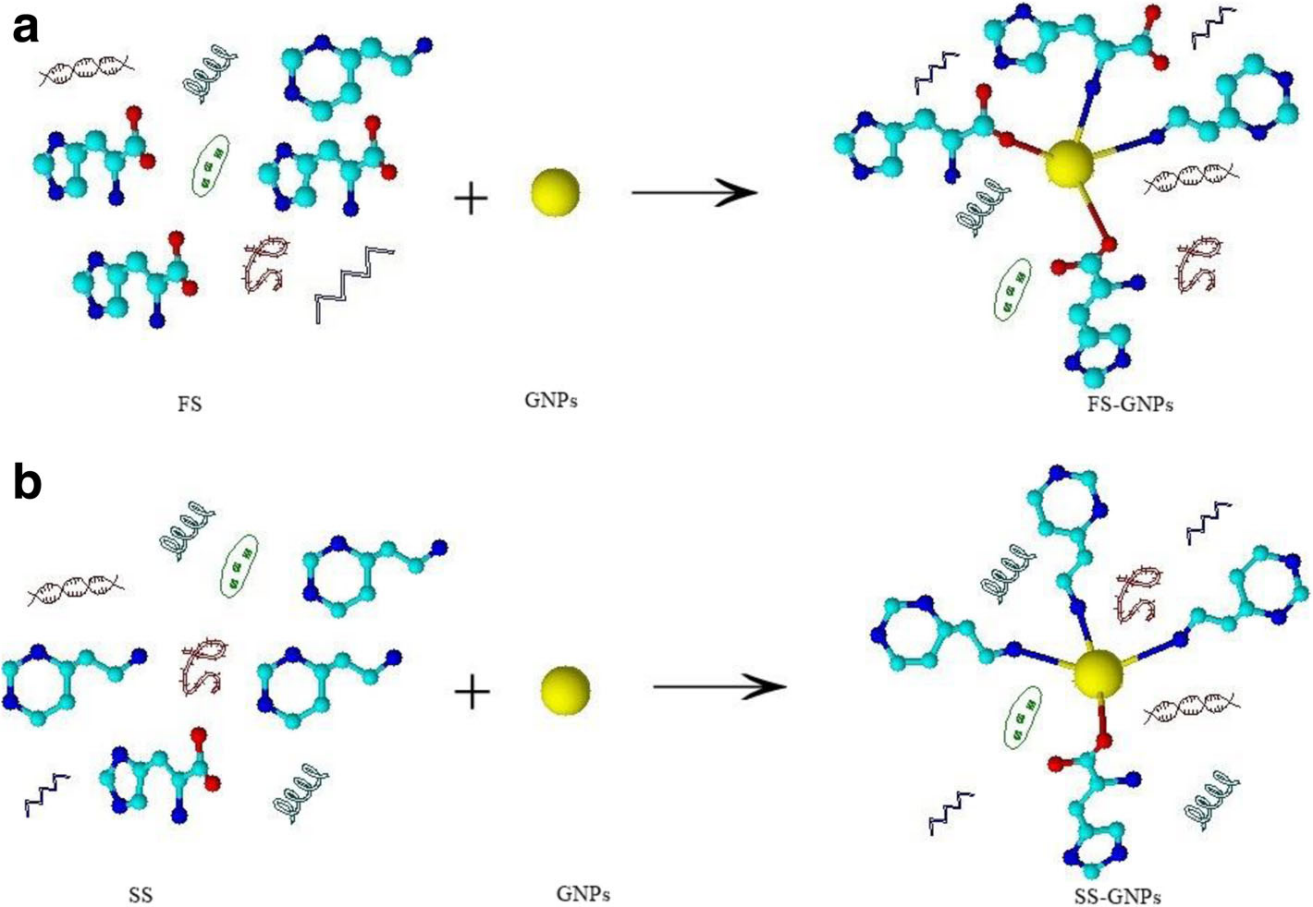
Supposed interaction between FS and SS with GNPs to form **a** FS–GNPs and **b** SS–GNPs: yellow = GNPs, red = O, blue = N, and cyan = C

So, according to the FTIR spectra, the reaction of FS and SS with GNPs could be summarized in Scheme 5.

### Comparison Between our Results and Other Results

Previously, a reversed-phase high-performance liquid chromatographic method is described for the quantitation of biogenic amines including histamine in chicken carcasses and detected by fluorescence [57]. This method was linear for the amines studied at concentrations ranging from 0.02 to 136 μmol/mL. Accuracy (recovery for histamine was 74.6%).

Another method using capillary zone electrophoresis (CZE) with conductometric detection of biogenic amines was also described [58]. Linearity by this method was (0–100 μmol/mL), and the accuracy (recovery 86–107%) and detection limit (2–5 μmol/L) were evaluated by this method.

Manju et al. [59] applied a method for the determination of histamine and histidine by capillary zone electrophoresis with lamp-induced fluorescence detection. The linear range was observed from 45 to 105 μM/mL. It showed a limit of detection of 48.7 μM and a limit of quantification of 132.4 μM. Accordingly, it is obvious that our method offers a rapid and sensitive histamine determination. Our measured sensitivity is 3 × 10^−3^, the linear dynamic range is from 2 to 16 μM with a limit of detection (LOD) of 0.6 μM. Because of the large enhancement of the surface electric field on the GNPs surface, the plasmon resonance absorption has an absorption coefficient orders of magnitude larger than the strongly absorbing dyes. Different sizes and shapes of GNPs have plasmon resonance absorptions that are even stronger, leading to increased detection sensitivity.

Chemically bonded molecules can be detected by the observed change they induce in the electron density on the surface, which results in a shift in the surface plasmon absorption maximum. This is actually why GNPs are used as sensitive sensor.

The easy and rapid steps of measurements of our method are considered as important factors comparing with the long and tedious preparation steps of the other reported methods.

## Conclusions

In this study, the sensing sensitivity of GNPs for measurements of histidine as a natural occurring amine found in chicken meat protein and histamine, resulting from bacterial decarboxylation of histidine, leading to denaturation of the protein signaling chicken meat spoilage is measured.

UV–visible, fluorescence, FTIR, and transmission electron microscopy (TEM) were used for characterization as well as the sensitivity measurements of histidine–GNPs and histamine–GNPs. Histidine-GNP and histamine–GNP sensitivities were found to be 7.70 × 10^−4^ and 6.59 × 10^−4^, respectively. Also, the LOD of both histidine and histamine in real fresh and spoiled chicken sample was detected using GNPs as a biosensor and was found to be 0.6 μM with a good sensitivity of 3 × 10^−3^ and a correlation coefficient of 0.993. The observed linear dynamic range of histamine is from 0.6 to 12 μM.

## Additional file

Additional file 1: UV–vis spectra of GNPs, histidine–GNPs, histamine–GNPs, FS–GNPs, SS–GNPs. (DOCX 76 kb)
