# Supplementary figures and images for: Gold Nanoparticles as a Direct and Rapid Sensor for Sensitive Analytical Detection of Biogenic Amines
Source: Nanoscale Res Lett. 2017 Mar 29;12:231. doi: 10.1186/s11671-017-2014-z (PMC5371533; doi:10.1186/s11671-017-2014-z)

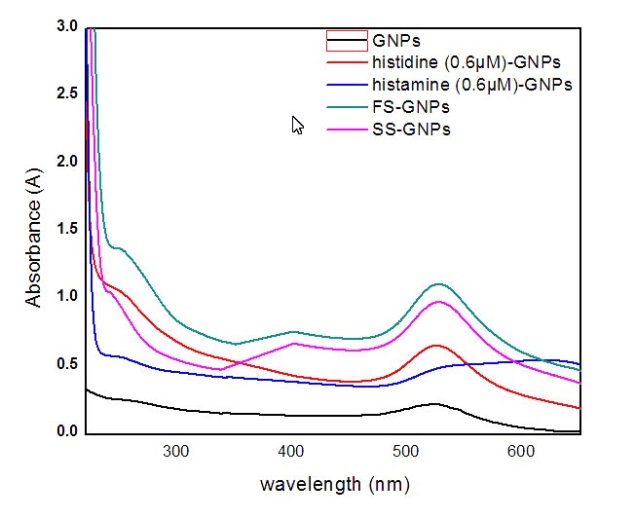


UV-vis spectra of GNPs, histidine-GNPs, histamine-GNPs, FS- GNPs, SS-GNPs

Supplement: Additional file 1: — UV–vis spectra of GNPs, histidine–GNPs, histamine–GNPs, FS–GNPs, SS–GNPs. (DOCX 76 kb) [file 11671_2017_2014_MOESM1_ESM.docx]
